# IGF-1R and Leptin Expression Profile and the Effects of Metformin Treatment on Metabolic and Endocrine Parameters in PCOS Mice

**DOI:** 10.1155/2017/9058307

**Published:** 2017-12-21

**Authors:** Luis Eduardo Prado Correia, Bruna Cristine de Almeida, Manuel de Jesus Simões, Mauro Abi Haidar, Daniela Berguio Vidotti, Ivaldo Silva

**Affiliations:** ^1^Climacteric Section, Department of Obstetrics and Gynecology, Universidade Federal de São Paulo Escola Paulista de Medicina (UNIFESP/EPM), 66 Embaú Street, Vila Clementino, 04039-060 São Paulo, SP, Brazil; ^2^Laboratory of Molecular and Structural Gynecology, Department of Obstetrics and Gynecology, University of São Paulo School of Medicine, 455 Dr. Arnaldo Avenue, Room 4121, Cerqueira Cesar, 01246-903 São Paulo, SP, Brazil; ^3^Histology and Biology Structural Division, Morphology Department, Universidade Federal de São Paulo Escola Paulista de Medicina (UNIFESP/EPM), 740 Botucatu Street, Vila Clementino, 04023-009 São Paulo, SP, Brazil; ^4^University Center of United Metropolitan Colleges, 1239 Santo Amaro Avenue, Vila Nova Conceição, 04505-001 São Paulo, SP, Brazil

## Abstract

We aim to assess the effects of metformin treatment on metabolic and endocrine parameters and genes expression related to the insulin-responsive pathway in polycystic ovary syndrome (PCOS). This study comprises twenty-eight obese mice divided into three metformin-treated groups for seven and twenty days and eight nonobese and nontreated ones. We found a significant decrease in glycemia after metformin treatment at days seven and twenty. However, we did not observe differences in body weight measurement. Histologically, after twenty days we observed follicular development with regression of androgenic effects. Levels of IGF-1R protein expression were low after twenty days of treatment, but LEP proteins showed an overexpression in the ovarian stroma. We assessed the IGF-1R and LEP mRNAs levels; data showed a significant overexpression of LEP after seven days of treatment, while the IGF-1R was downregulated. Metformin therapy seems to exert a beneficial effect on histological and anovulatory features, reducing follicular number and pyknosis formation, possibly involved in the reversion of androgenic stimulus. Expression of IGF-1 and LEPR indicates a relevant role in androgenic features reversion present in PCOS, hormonal equilibrium, body weight regulation, and glucose metabolism, therefore, under phenotype obesity and infertility regulation in this model.

## 1. Introduction

Polycystic ovary syndrome (PCOS) has been considered the most common multifactorial endocrinopathy syndrome [1, 2], which affects about 15% of reproductive age women [2, 3]. It is a heterogeneous condition characterized by chronic anovulation, hyperandrogenism [4, 5], and polycystic ovarian morphology [6]. PCOS etiology is still not totally understood.

Ovarian hyperandrogenism in PCOS may arrest follicle genesis through inhibition of granulosa cell proliferation and maturation, estrogen and progesterone secretion, aromatase action, and increase of 5a-reductase activity [7]. Hyperandrogenism associated with obesity induces cardiometabolic dysfunction and chronic low-grade inflammation. When associated with insulin resistance (IR), it appears to play a pivotal role in the PCOS pathophysiology [2, 8] by influencing both growth and function of ovarian cell [9]. IR accompanied by compensatory hyperinsulinemia is a common feature of PCOS and some evidences suggest that hyperinsulinemia plays a pathogenic role in causing the hyperandrogenism of the syndrome by increasing ovarian androgen production [10]. Of note, hyperandrogenism and IR in families of PCOS patients suggest that PCOS might have a genetic origin [11, 12].

Genes related to insulin signaling pathways, which encode steroidogenic enzymes and regulate androgen biosynthesis involved in insulin secretion and action, such as insulin-like growth factor-1 (IGF-1R) and leptin (LEP) have been widely studied in an attempt to elucidate PCOS dysfunction [12].

Genomically, the IGF-1R is a membrane-bound receptor which is capable of autophosphorylation following ligand binding. IGF-1 is the major ligand for this receptor, but both IGF-2 and insulin can bind to the IGF-1R. Following ligand binding and IGF-1R autophosphorylation, insulin receptor substrate proteins are tyrosine phosphorylated by the IGF-1R. Dysregulation of the IGF-1/insulin/IGF-1R system may contribute to the pathophysiology of premature adrenarche and PCOS [13]. IGF-1R has the capacity to stimulate, through its own receptor, androgen production by ovarian cells [14]. This protein was identified in antral and preantral follicles [15]. Evidence suggested that ovarian hyperandrogenism is a result of insulin action on the ovaries and because of that, it is mediated by IGF-1R [14].

Another factor that amplifies the clinical severity of PCOS is obesity [8]. Metabolic dysfunctions that are obesity-related may be associated with dysregulated expression of LEP, which may also have a role in reproductive function, acting at many levels of the hypothalamic-pituitary-ovarian axis [3]. This hormone peptide secreted from adipose tissue plays an important role in food intake and energy homeostasis [6]. Clinical trials demonstrated that elevated leptin levels are detected in women with PCOS compared with non-PCOS [16]. A negative correlation between insulin sensitivity and LEP levels was seen in obese and nonobese PCOS patients [17]; insulin may act as a stimulant of LEP gene expression, enhancing leptin secretion [6]. Moreover, higher LEP levels may be correlated with insulin receptor, metabolic disorder, infertility, and even cardiovascular disease risk in PCOS, which may contribute to the etiology and development of PCOS [16].

Treatment approach to PCOS generally focuses on goals for insulin-sensitizing drugs such as metformin to treat infertility and a chronic therapy to prevent long-term consequences of PCOS [18]. Metformin is considered the first-line treatment for ovulation disorders and infertility [19]; it is commonly used in PCOS for providing ovulatory menstrual cycles and to increase the rate of clinical pregnancy in PCOS patients, besides optimizing the effects of other agents of ovulatory induction in patients with clomiphene citrate resistance [20]. Metformin reduces body weight and improves hyperandrogenism, reproductive function, and metabolic parameters such as hypertension, hyperlipidemia, and glycemic control in women with PCOS [18, 19, 21].

Little is known about the role of IGF-1R and LEP in the metabolic dysfunctions, as well as the action of metformin in PCOS. Elucidation of the metformin treatment in the metabolic system and the action of the genes and their insulin-related proteins may be helpful to develop novel therapies and in diagnosis for PCOS patients. We aimed to assess the effects of metformin treatment on metabolic and endocrine parameters, as glycemia and body weight, ovarian morphology and physiology, and the IGF-1R and LEP expression in PCOS.

## 2. Materials and Methods

This study was carried out in the Gynecology Molecular and Proteomics Laboratory and in the Histology and Biology Structural Laboratory of the Federal University of São Paulo (UNIFESP/EPM), from 2011 to 2014, and it was approved by the Research Ethics Committee of the Federal University of São Paulo (UNIFESP/EPM) and registered under code 0304/11.

Twenty-eight obese and eight nonobese, 8- to twelve-week-old female C57BL6, leptin-deficient (B6.Cg-m+/+* Lep*^*ob*^/J,* ob/ob*) and leptin receptor mutant (B6.V-*Lep*^*ob*^/J,* ob/ob*) mice were housed in the Development of Experimental Models of Medicine and Biology Center (CEDEME), UNIFESP/EPM. Rooms provided a controlled temperature range of (22–24°C) on a fourteen-hour light and ten-hour dark cycle. Animals were weighed before treatment. Models were assigned to four different groups and treated for seven and twenty days, as follows:

1. Control group: nonobese B6.V-Lep^*ob*^/J mice (*n* = 8) without treatment with normal glycemia and ovulation
2. Obese group: overweight B6.V-Lep^*ob*^/J,* ob/ob* mice (*n* = 9) without treatment that develops hyperglycemia and anovulatory
3. Obese plus metformin 7 (Met 7): overweight B6.V-Lep^*ob*^/J,* ob/ob* mice that develop hyperglycemia and anovulatory, treated with 50 mg/kg (milligram/kilogram) of 500 mg metformin (Medley, USA) daily (metformin was added to 0.05 mL (milliliter) water and orally administered (*n* = 7); at the end of the eighth day, the animals were euthanized).
4. Obese plus metformin 20 (Met 20): overweight B6.V-Lep^*ob*^/J,* ob/ob* mice that develop hyperglycemia and anovulatory, treated with 50 mg/kg of 500 mg metformin (Medley, USA) daily (metformin was added to 0.05 mL water and orally administered (*n* = 12); at the end of the twenty first day, animals were euthanized).

### 2.1. Blood Glucose Measurement

Subsequently, animals were weighed and sacrificed and the blood was collected from the caudal artery, and the blood glucose levels were measured with blood glucose test strips ACCU-CHECK® Sensor (CA, USA).

### 2.2. Real-Time PCR Array

Total RNA was extracted from frozen ovarian tissue with TRIzol® Reagent (Ambion™, NY, USA) according to the manufacturer's instructions. Subsequently, the reverse transcriptase (RT) was performed using the RT^2^ First Strand Kit (Ref: 330404; Qiagen, Hilden, Germany); and the quantitative Real-Time PCR array was carried out using the RT^2^ Profiler™ PCR Array Mouse Insulin Signaling Pathway (PAMM-030A; Qiagen, Hilden, Germany), 96-well plate, and RT^2^ SYBR® Green qPCR Mastermix (Qiagen, Hilden, Germany). The reaction conditions were as recommended by the manufacturer, and all assays were performed in triplicate. All data were normalized with the* Gusb*,* Hprt*,* Hprt1*,* Hsp90ab1*,* Gapdh*, and* ACTb* genes and analyzed by ΔΔ*C*_*t*_ method in the http://pcrdataanalysis.sabiosciences.com/pcr/arrayanalysis.php software, following the manufacturer's instructions.

### 2.3. Conventional Histopathological Method

Ovaries were dissected and immediately fixed in 10% formalin for histological assessment and processed for paraffin embedding. Formalin-fixed and paraffin-embedded tissue blocks were cut into serial sections (5 micrometers) using an AO American Optical 820 Rotary Microtome (AO Instrument Company, NY, USA). Briefly, after assembly into a glass slide, the tissue was deparaffinized in xylene, rehydrated in graded alcohols (100%, 95%, and 70%) for 5 minutes each, and stained with hematoxylin for 10 minutes and eosin for 7 minutes (HE staining) followed by sealing with Entellan® (Merck Millipore, Darmstadt, GE).

### 2.4. Immunofluorescence Staining

All groups of ovarian tissue were processed using immunofluorescence. After deparaffinization and rehydration, ovarian tissues from each group were fixed in 4% paraformaldehyde in PBS at 4°C for 20 minutes, followed by permeabilization in 0.1% Triton X-100-PBS (pH 8.2) (Sigma-Aldrich, MO, USA). Nonspecific binding sites were blocked with Protein Block Serum-Free (DakoCytomation, Denmark) in darkness for 2 hours and incubated with the IGF-1R polyclonal antibody (anti-Rabbit, 1 : 200; Cell Signalling, MA, USA) and LEP polyclonal antibody (anti-Rabbit, 1 : 150; Biogen, USA) overnight at 4°C.

For all instances, replacing the primary antibody with 0.1% Triton X-100-PBS (Sigma-Aldrich, MO, USA) and adding only secondary antibody for 1 hour at room temperature in darkness were used as a negative control to confirm the specificity of labeling. The secondary antibody used was anti-Rabbit Rhodamine- (TRITC-) labeled IgG (1 : 700; Abbiotec, CA, USA) for both IGF-1R and LEP. The slides were then covered with mounting medium for fluorescence with DAPI (4′,6-diamidino-2-phenylindole dihydrochloride; Vector Laboratories, CA, USA) and the fluorescence images were obtained with Olympus BX51 (Olympus Corporation, Tokyo, JP) at magnification ×100 and ×400.

### 2.5. Statistical Analysis

Statistical tests were performed using GraphPad Prism 3.00 (GraphPad Software, San Diego, CA, USA) for Windows. Continuous data were described as mean and standard error of mean (SEM). All data were normally distributed. Difference across groups was analyzed by Student's *t*-test and ANOVA test. Statistical significance was established as *P* < 0.05.

## 3. Results

**Figure 1:**
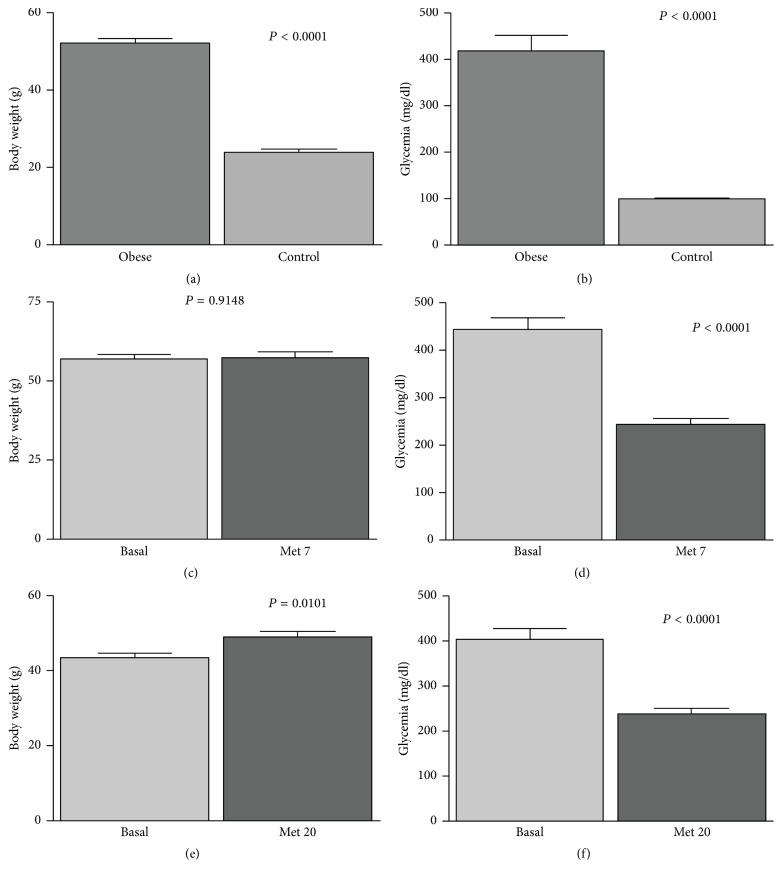
Between-subject effects for variables body weight and glycemia in two groups of metformin treatment (B6.V-Lep^*ob*^/J,* ob/ob* mice with PCOS). Gram: g; milligrams/deciliter: mg/dl.

First, we assessed body weight (g) and glycemia (mg/dl) measurements in the control and obese groups without treatment (Figures 1(a) and 1(b)). Regarding body weight evaluation (mean ± SEM), obese group (52.0 ± 1.3) showed statistical significance compared with control group (24.0 ± 0.7). Glycemia was statistically relevant in comparisons between obese (418.4 ± 33.0) and control groups (100.2 ± 2.2).

Body weight of Met 7 group was evaluated before the beginning of metformin treatment; basal value (57.1 ± 1.4) was compared with measurement after seven days of treatment (57.4 ± 1.6) as showed in Figure 1(c). An association of glycemia reduction in the Met 7 group (243.4 ± 12.2) was observed (Figure 1(d)). Metformin treatment during twenty days leads to a decrease in glycemia in Met 20 group (240.2 ± 13.3), but difference in body weight was not observed (Figure 1(f)).

**Figure 2:**
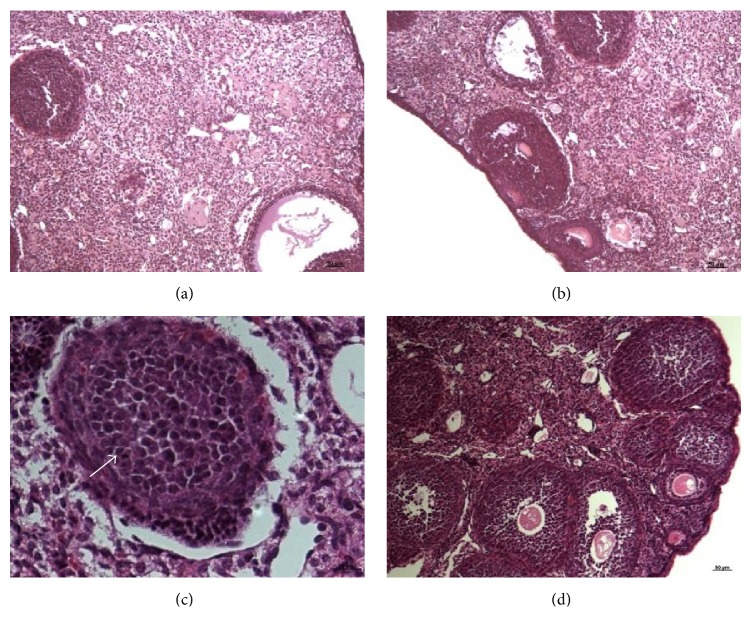
Microphotographs showing the HE staining of B6.V-Lep^*ob*^/J,* ob/ob* mice with PCOS. (a, b) Met 7 group ×10; (c) Met 20 group (magnification ×40) and (d) Met 20 group (magnification ×10).

Histological sections of ovaries with HE staining after seven days of treatment, Met 7 group, showed a similar pattern with control group exposing the presence of several degenerate follicles, abundant interstice, and absence of corpus luteum (Figures 2(a) and 2(b)). In the daily treatment with metformin during twenty days, Met 20 group showed a reducing number of follicles in all phases of development, including preantral, antral, and advanced phases of development, along with the blood vessels and corpus luteum. We noticed the intense formation of pyknosis presenting a reversal of androgenic stimulus and starting a greater formation of follicles after the metformin use (Figures 2(c) and 2(d)).

### 3.1. mRNA Expression of Insulin-Related Genes

**Figure 3:**
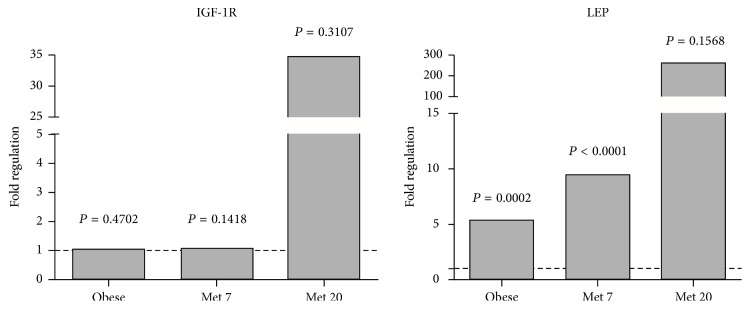
Genetic expression of IGF-1R and LEP genes insulin-related pathway. The three groups were compared with control group.

We assessed the expression profile of 84 genes related to the insulin-responsive pathway. Some genes had expression changes along the treatment. Short-term treatment, during seven days, seemed to have a beneficial effect in the reversal of mRNA expression levels of genes related to insulin pathway. This profile of genetic expression was not observed in the prolonged treatment during twenty days. Prolonged metformin use might lead to an enhancing effect on the genetic expression, overexpressing some relevant genes in this signaling pathway. Met 20 group showed an increase of expression mainly in the IGF-1R and LEP genes (Figure 3).

### 3.2. Protein Expression of IGF-1R and LEP

**Figure 4:**
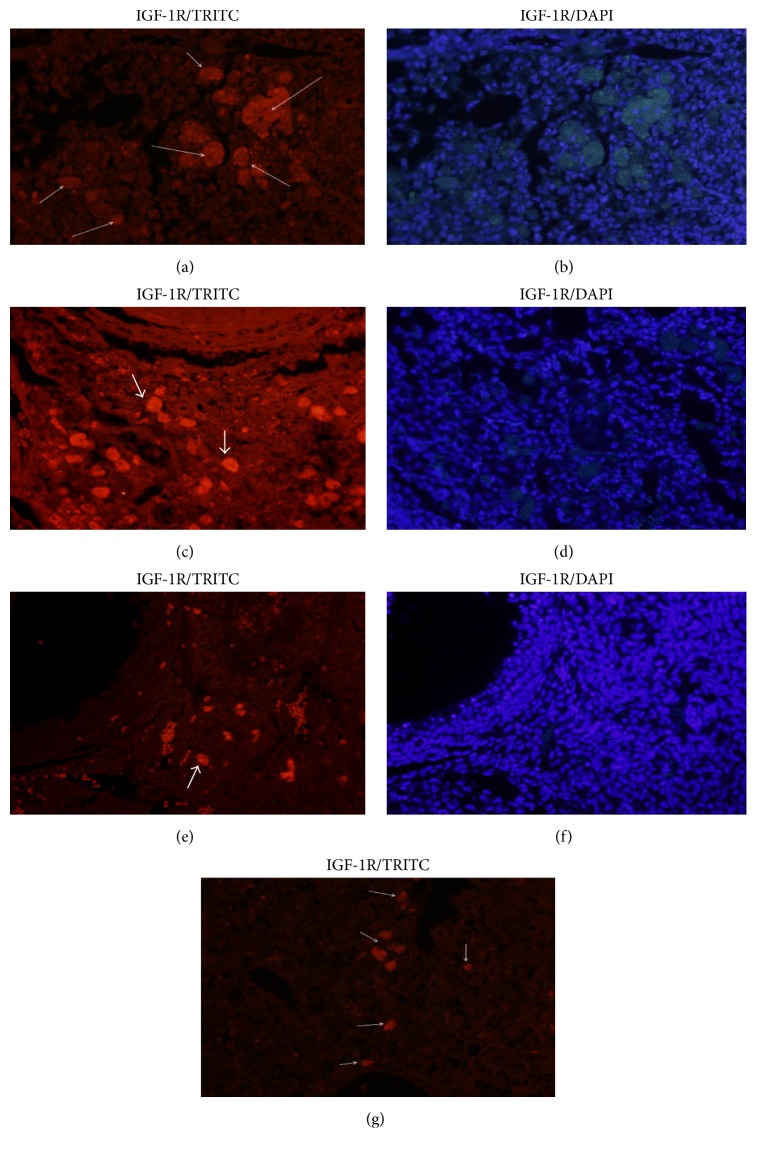
IGF-1R protein expression in ovarian tissue sections in different treated groups. (a) The obese nontreated group with TRITC and (b) obese nontreated group with DAPI. (c) Met 7 group with TRITC and (d) Met 7 group with DAPI. (e) Met 20 group with TRITC and (f) Met 20 group with DAPI. (g) Control group with TRITC (magnification ×40).

Low expression of IGF-1R protein was identified in the ovarian stromal tissue in control group and an increased expression was observed in the obese nontreated group (Figures 4(a) and 4(b)). After metformin use during seven days, we observed a smaller amount of protein spreader in ovarian stromal cells compared with obese nontreated group (Figures 4(c) and 4(d)). Decrease of IGF-1R protein expression may be seen after twenty days of treatment with an expression profile similar to control group (Figures 4(e) and 4(f)).

**Figure 5:**
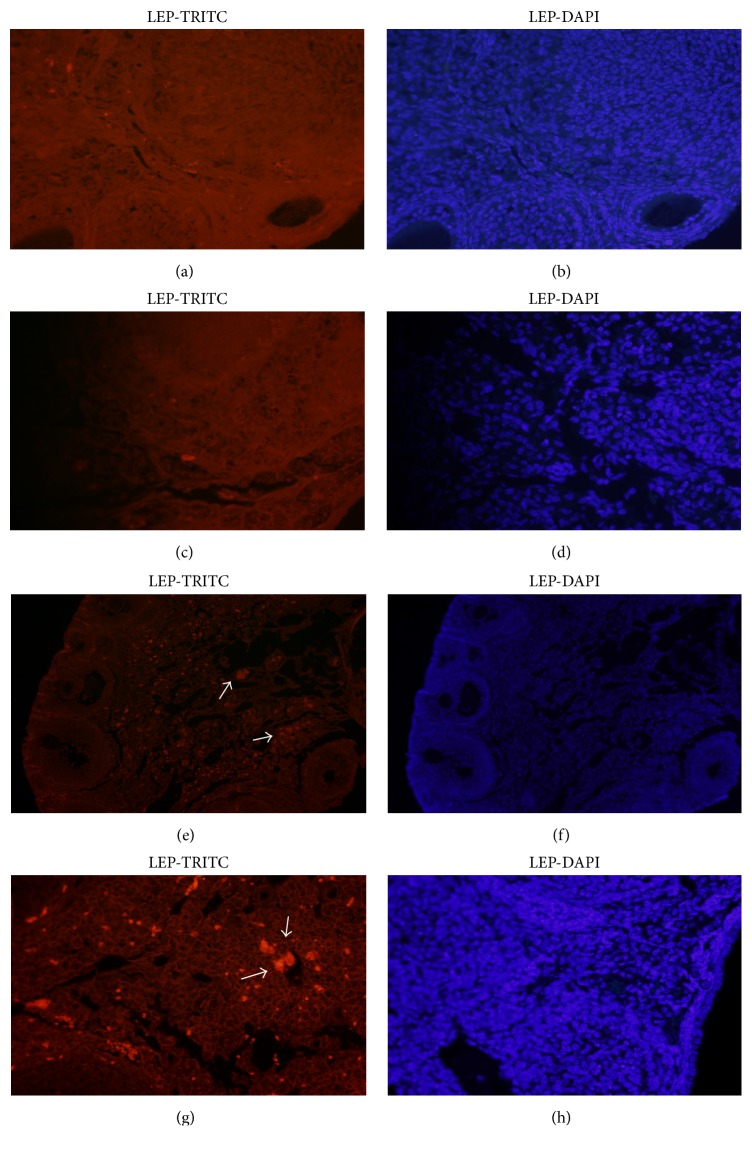
LEP protein expression in ovarian tissue sections in different treated groups. (a) Control group with TRITC and (b) control group with DAPI. (c) Obese group nontreated with TRITC and (d) obese group nontreated with DAPI (magnification ×40). (e) Met 7 group with TRITC and (f) Met 7 group with DAPI (magnification ×10). (g) Met 20 group with TRITC and (h) Met 20 group with DAPI (magnification ×40).

Subsequently, we evaluated the LEP expression in the ovarian stroma. Control group confirmed the almost nonexistent cell structure marking, which showed the relevance of this gene in the reversal of androgenic effects in obese cases (Figures 5(a) and 5(b)). Our data indicated that after seven days of metformin administration an increase of LEP expression occurred (Figures 5(e) and 5(f)). We noticed that there was a great amount of LEP proliferation and the expression of leptin is spread in the ovarian stroma (Figures 5(g) and 5(h)).

## 4. Discussion

Animal model proposed in our study showed that obese mice have similar PCOS features including obesity, innumerable ovarian polymicrocysts, chronic anovulation, and infertility besides constant hyperglycemia.

Particularly, the control and obese groups showed glycemia variation, but we have not found differences in the body weight. After the metformin treatment for seven days, we have found a significant reduction in glycemic levels. Use of metformin in low dose (0.2–5 mg/mL in water) during six weeks in mice, for glycemic control, may lead to an important decrease of glycemic values [22], which corroborates with our findings. Given the pivotal role IR and obesity play in the etiopathogenesis and progression of PCOS and its potential subsequent metabolic and cardiovascular complications, both, should be considered essential therapeutic targets [23].

Ovarian morphology of PCOS includes the increase in the number of follicles, the density of the stroma, and the volume of the ovary itself. Furthermore, an overall increase in the pulsatility index of the uterine arteries may occur [24]. According to their size, antral follicles were classified into two categories. The first is represented by smaller follicles with high circulating androgen concentrations, which are significantly raised in PCO compared with normal; the second category encompasses larger follicles, which reflects the degree of IR, and it is closely linked to the frequency of follicular maturation, ovulation, and infertility. These follicular categories represent later stages of development before the preovulatory stage. Thus, the rates of initial recruitment and progress through development stages appear to differ from normal [5]. In our histological sections, the ovaries of obese mice had no corpus luteum development, but there was the presence of follicles and interstitial cells comparing with controls. Metformin-treated mice presented degenerate follicles, abundant interstitium, and absence of corpus luteum after seven days of treatment. These ovaries resemble obese group without treatment.

Very relevant differences on the metformin twenty-day treated group were found once there was a turnaround on the compatible characteristics with anovulation, including reduced numbers of follicles in every phase of development, such as preantral, antral, and advanced stages of it, besides blood vessels. A significant amount of pyknosis could be seen, showing then “androgenic” trigger reversal, features that were significantly different compared with seven-day treated mice and nontreated obese mice. The use of metformin for twenty days was very efficient in the glycemia control, as we had a significant glycemia drop when compared with the nontreated obese group. Omran [5] asserted that the metformin induces normalization of ovarian, follicular, and corpus luteum vascularization. In addition, it can also improve several surrogate markers of endometrial receptivity, that is, uterine, subendometrial, and endometrial blood flow and endometrial thickness and pattern. Several studies have pointed out that metformin is an effective treatment for anovulation in women with polycystic ovary syndrome and leads to a relevant reduction in the insulin levels [25–27], and metformin isolated or associated with other drugs might lead to ovulation return reducing the PCOS features [28–35]. Heishi et al. [36] reveled that after two hours of 400 mg/kg metformin administration in mice the first relevant metformin action was the hepatic gluconeogenesis decreases followed by the increase of peripheral insulin sensitivity.

Remarkably, women with PCOS have normal insulin molecules and the insulin receptor on cells appears to be normal. However, it appears to be a postreceptor deficit, in relation to the downstream cellular effects of what happens after insulin binds to the insulin receptor, meaning that the molecular cascade of intracellular events has a level of impairment, leading to a postreceptor “intracellular” resistance to insulin [37]. Thereby, the most important finding in our study was 14 genes with reduction of expression, including the glucose-6-phosphatase gene and genes linked to glucose and lipid metabolism. Some genes seem to influence and/or to intensify the PCOS symptoms. So, we selected the IGF-1R and LEP genes to investigate the expression levels and their roles in PCOS, seeking to elucidate the effects of metformin treatment on gene expression.

Although IGF-1R expression did not show significance, we noticed that the treatment with metformin for seven days kept the levels of gene expression similar to control group; however, after twenty days, the metformin seemed to overexpress the IGF-1R gene. Prospective studies may be designed in large-scale PCOS populations that might identify the causal effect of IGF-1R expression in long-term therapy.

LEP showed a significant overexpression in the obese and Met 7 groups. Against this background, the role of LEP has been subject to profound controversy, with opposing views regarding its true participation. Because LEP concentrations are consistently found to be strongly correlated with weight, some reports considered the hyperleptinemia seen in PCOS as only a byproduct of this condition [23, 38].

We also observed alterations in the expression in nine genes between the study groups, Cfd, Frap1, Insl3, Pck2, Ppp1ca, PrKci, Shc1, and Slc2a1 related to the insulin-responsive pathway. The Cfd was downregulated in the obese group while Frap1, Insl3, Pck2, Ppp1ca, PrKci, Shc1, and Slc2a1 were overexpressed. Some genes suffered expression alterations after seven days of metformin treatment showing an expression closer to the control group; this may suggest a potential role of metformin as a regulator of the hyperinsulinemic environment. Although a short-term metformin therapy has been shown to be beneficial, the same was not observed for metformin administration during twenty days. In the treated group during twenty days, the metformin intensified the alteration of gene expression, but more studies are necessary to clarify the effects of long-term metformin therapy.

Variations of IGF-1R and LEP proteins expression were found in all groups. IGF-1R protein expression showed a significant increase of stromal immunostaining in the obese group and continuous alterations increase in the group treated with metformin for seven days. On the other hand, after a long time, the group treated for twenty days showed a normalization trend suggesting that the regulation of IGF-1R expression possibly occurs due to the therapy time. The protein family insulin growth factor (IGF) has similar actions to insulin on glucose metabolism* in vivo,* stimulating the peripheral glucose consumption and the use and reduction of hepatic glucose production [39]. De Leo et al. [26] emphasized that metformin reduces plasma insulin levels and IGF-I availability to the ovaries and this may modify the hyperandrogenic intrafollicular milieu recognized in PCOS.

Another interesting finding in the obese mice ovaries was the low immunostaining for LEP. On the seven-day treated group, we detected some LEP proteins expression in the ovaries tissue, and an increase of a LEP proteins expression was seen in the ovaries tissue with treatment for twenty days. Thereby, we can conclude that this stromal protein has a key role in the reversion of androgenic medium present in the obese mice ovaries. LEP exerts direct effects in all ovarian cells and seems to have a physiologic regulatory effect in folliculogenesis [23, 40].

Interestingly, studies where the findings link LEP levels to estradiol, testosterone, and insulin in women with PCOS advocate for a more complex role of LEP in its pathophysiology [23, 41, 42]. Reported data of elevated LEP in nonobese PCOS patients further question quantitative adiposity as the sole origin of hyperleptinemia in this scenario [23, 43].

Rojas et al. [23] affirm that hyperleptinemia exerts direct effects on ovarian physiology by arresting follicle development. Ovarian paracrine and autocrine LEP signaling may also be disrupted, parallel to alterations in the hypothalamus-hypophysis-ovary axis. Likewise,* in vitro* studies show a decrease in collagenase expression in ovarian tissue after exposure to high concentrations of LEP, adding to the ovulatory disturbances in this scenario.

## 5. Conclusion

In summary, the metformin short-term therapy (seven days) may reduce significantly glycemia, but body weight does not seem to suffer any metformin influence. Therapeutic effects were efficient in the reversion of histological alterations of the anovulation features and the decrease of follicles number, besides the considerable number of pyknosis formation, possibly involved in the reversion of androgenic stimulus. Normalization of stromal proteins expression of IGF-1R and LEP in obese mice ovaries may be essential for reversion of androgenic medium, hormonal equilibrium, in the body weight regulation and the glycose metabolism, and, therefore, the regulation of obese/infertility phenotype of this model.

Metformin is mainstay insulin sensitizer and traditionally used for PCOS treatment, but distinct phenotypes for this syndrome bring into question its indication in all cases of PCOS. Controversial data regarding treatment of this syndrome indicates how necessary are further studies to elucidate the intricacies within the pathophysiology of PCOS and the true relationship between IR, hyperinsulinemia, and hyperandrogenemia, as well as other important hormonal disturbances, alterations in steroid hormone metabolism, hyperleptinemia, and LEP resistance [23]. The results presented herein provide additional evidence that the simultaneous evaluation of IGF-1R and LEP immunoexpression could add information to the treatment strategy in PCOS patients. Nonetheless, more studies are necessary to further our understanding about these proteins in PCOS development and treatment, once great amounts of women are presenting this metabolic disorder that may result in infertility.
